# Big Changes Start With Small Talk: Twitter and Climate Change in Times of Coronavirus Pandemic

**DOI:** 10.3389/fpsyg.2021.661395

**Published:** 2021-06-15

**Authors:** Mariana Gaytan Camarillo, Eamonn Ferguson, Vanja Ljevar, Alexa Spence

**Affiliations:** ^1^Department of Psychology, University of Nottingham, Nottingham, United Kingdom; ^2^Business School, University of Nottingham, Nottingham, United Kingdom

**Keywords:** environmental psychology, coronavirus, actions, climate change, public perceptions, Twitter

## Abstract

Behavioural scientists have been studying public perceptions to understand how and why people behave the way they do towards climate change. In recent times, enormous changes to behaviour and people’s interactions have been brought about by the worldwide coronavirus disease 2019 (COVID-19) pandemic, unexpectedly and indefinitely; some of which have environmental implications (e.g., travelling less). An innovative way to analyse public perceptions and behaviour is with the use of social media to understand the discourse around climate change. This paper focuses on assessing changes in social media discourse around actions for climate change mitigation over time during the global pandemic. Twitter data were collected at three different points during the pandemic: February (time 1), June (time 2), and October 2020 (time 3). By using machine learning techniques, including recurrent neural networks (RNN) and unsupervised learning Latent Dirichlet Allocation (LDA) topic modelling, we identified tweets mentioning actions to mitigate climate change. The findings identified topics related to “*government actions*,” *“environmental behaviours*,” “*sustainable production*,” and “*awareness*,” among others. We found an increase in tweets identified as “action tweets” relating to climate change for time 2 and time 3 compared with time 1. In addition, we found that the topic of energy seemed to be of relevance within the public’s perceptions of actions for climate change mitigation; this did not seem to change over time. We found that the topic of “*government actions*” was present across all time points and may have been influenced by political events at time 1, and by COVID-19 discourse at times 2 and 3. Moreover, topic changes over time within Twitter indicated a pattern that may have reflected restrictions on mobility as these tended to focus on individual and private sphere behaviours rather than group and public sphere behaviours. Changes in topic patterns may also reflect an increase in salience of certain behaviours (e.g., shopping), which may have received increased attention due to lockdown restrictions. Considering restrictions and adaptability challenges people face in times of a global pandemic may help to identify how to support sustainable behaviour change and the likely persistence of these changes.

## Introduction

Climate change is one of the major global challenges for society. The study of how the public perceives climate change and factors influencing mitigation and adaptation behaviours are important to respond effectively to this environmental issue. The use of big data and social media platforms such as Twitter to study climate change discourse is a relatively novel approach. Social media constitutes a vast pool of data available for the analysis and understanding of public opinions regarding climate change (Wu et al., 2016; Veltri and Atanasova, 2017; Pearce et al., 2019). With the emergence of the coronavirus disease 2019 (COVID-19) pandemic, the behaviour of individuals has faced challenging changes in different domains (e.g., health, social interaction, and travelling), some of these changes are followed by both positive (e.g., emission reductions) and negative impacts (e.g., putting aside environmental priorities; Helm, 2020). Recent research on COVID-19 has suggested that disruptive changes to people’s behaviours have had an impact on the environment in the form of reduction of emissions, air pollution, and free movement of wildlife (Helm, 2020; Rupani et al., 2020). In addition, the impact of coronavirus and the regulations to control it have had an effect on the public perceptions of regulations and policies needed for climate change (Bostrom et al., 2020). However, to date, there is no evidence on how the discourse on actions undertaken intentionally to mitigate climate change has been impacted by COVID-19.

In 2020, the WHO declared a public health emergency as a consequence of COVID-19 and warned that restrictions on mobility and confinements might negatively impact physical health and psychological well-being (World Health Organization, 2020). The global pandemic and its impact on different aspects of individuals’ behaviour have been the focus of many researchers throughout the year, studying the widespread anxiety and considerable uncertainty as the security of jobs is challenged by the pandemic (Office for National Statistics, 2020), as well as the increase of domestic violence during lockdown (Bradbury-Jones and Isham, 2020) among other things.

In terms of the impact of COVID-19 on the environment, research showed some positive changes in the short term such as the drop in carbon emissions, a sharp decline in transport use and, with it, the burning of oil (Helm, 2020); decrease of water pollution (Saadat et al., 2020); levels of air pollution falling as a result of countries’ efforts to combat the virus (Rupani et al., 2020); more wildlife movement in people-dominated areas; and a decrease in wildlife deaths on the streets (Shilling and Waetjen, 2020). Among the negative impacts of COVID-19 that have been identified are the increase in demand for plastic for healthcare usage (e.g., gloves, masks, and disposable plastic items; Klemeš et al., 2020); and an increase of domestic waste due to lockdowns (You et al., 2020). In addition, the coronavirus has consumed a vast amount of political and administrative bandwidth, putting environmental priorities aside (Helm, 2020).

Bostrom et al. (2020) evaluated public understanding of climate change and coronavirus threats, and how people’s perceptions of each influenced their level of related concern and willingness to act to tackle these threats. The researchers found that individuals perceived these two risks to be very similar and that learning about the actions to control the pandemic may have a spillover effect on people’s perceptions of climate change and the need for implementation of environmental policies. However, the researchers’ findings also suggested that the threat of climate change was also perceived as less worrying in comparison with the coronavirus pandemic (Bostrom et al., 2020); these results may be due to the relative novelty of the consequences of the pandemic in comparison with the enduring concerns of climate change. The authors considered that an effect of a “worry budget” may help explain their findings; the idea that people have a limited cognitive capability for worry, which allows them to worry about one thing at a time (Achen and Bartels, 2017; Bostrom et al., 2020).

The experience of the pandemic has also raised the prominence of the debate of health issues and their link with climate change. As a result of the impact of the coronavirus on people’s lives, the researchers suggest that a change in individuals’ perceptions of actions for climate change mitigation could occur, reducing the enthusiasm for globalisation (Helm, 2020), and promoting the search for cleaner sources of energy. Researchers have suggested that the mortality rate associated with COVID-19 could be linked to the poor quality of the air, ozone, and nitrogen oxides (Travaglio et al., 2020). How peoples’ perceptions of what needs to be done to mitigate climate change have varied in times of a global pandemic has yet to be examined systematically.

Public perceptions of climate change are important in understanding how people engage with climate change mitigation. The Committee on the Human Dimensions of Global Change considers people’s perceptions of global phenomena, such as climate change, a crucial contributor to the understanding of environmental problems and a determining factor in the development of possible solutions (Stern et al., 1997). Individuals’ perceptions of climate change and their willingness to support actions around climate change are dependent on context and individual experiences (Marshall et al., 2005; Weber, 2006; Dessai and Sims, 2010); availability heuristics (Tversky and Kahneman, 1974); and political polarisation (Weber, 2010; Kahan et al., 2011; Poortinga et al., 2019). There is also evidence of differences in climate change perceptions between scientists and the public in general. In 2009, a Pew research centre poll reported that while 85% of scientists agreed that the earth is getting warmer due to anthropogenic activity (e.g., burning fossil fuels), only 49% of the general public agreed with this statement (Kohut, 2009). This demonstrates the importance of understanding public perceptions of climate change mitigation in developing environmental communications and policies that are likely to be accepted and engaged with.

Public perceptions of climate change are typically measured through surveys such as the European Social Survey (ESS). Poortinga et al. (2019) used this survey on participant samples from 23 European countries to examine public perceptions of climate change and potential differences between countries. Findings indicated that sociopolitical and demographic factors had a clear impact on climate change beliefs, although this may be at least partially explained by differences in experiences people have with extreme weather events (e.g., flooding and droughts), and how vulnerable countries can be to the impacts of climate change (Brody et al., 2008; Spence et al., 2011; Deryugina, 2013; Demski et al., 2017). In addition to cross-cultural differences that may shape public perceptions of climate change, the differences in the ways that countries have experienced the global pandemic could influence perceptions of climate change and potential actions considered to mitigate it.

In contrast to examining public perceptions directly through surveys, there is now burgeoning research focusing on the analysis of public perceptions using naturalistic data freely available on social media. By 2019, of the 7.7 billion people in the world, 3.5 billion were reported to be online (Ortiz-Ospina, 2019). The increase in the use of social media and the disruption of traditional hierarchical structures of communication have weakened large media companies, political parties, and research organisations, while increasing the power of individuals to reach a mass of people through microblogs (Pearce et al., 2019). As a result, scientists have the availability of large amounts of data of people interacting in all sorts of ways, creating opportunities for the study of human behaviour.

The use of social media and big data tools has proved to make the gathering of large datasets more cost-effective, contributing to areas such as gambling and sports (Schumaker et al., 2016; Bradley and James, 2019); stock markets (Rao and Srivastava, 2014); and public mood (Bollen et al., 2011). Twitter is a platform structured as a microblogging website due to its character restrictions – 280 characters per tweet – users are forced to communicate information through reduced messages, sometimes making use of keywords, also known as “hashtags” (#), used to highlight the topic of their messages (Kirilenko and Stepchenkova, 2014).

Data from Twitter are theorised to have three main features (Veltri and Atanasova, 2017). First, the information is selected and displayed according to the user’s personal criteria and perceived relevance (instead of following journalistic criteria). Second, information is addressed to a particular audience made of network ties (rather than being broadcast to an unknown mass audience); and, lastly, information is often displayed in a conversational way rather than unidirectional (Veltri and Atanasova, 2017). This means that, on Twitter, discussions around one topic are driven by what the users perceive as relevant for their audiences.

Social media can also shape public awareness of social matters such as climate change. By disrupting the hierarchies of mass communication, social media has enabled all individuals to share any kind of information with thousands of people, regardless of the nature or veracity of this information (Brossard, 2013; Auer et al., 2014). Pearce et al. (2014) used Twitter hashtags to assess the dynamics of tweets mentioning the 2013 IPCC report, examining the role played by influencers in reaching different types of public. Findings suggested that, in general, people are more likely to have conversational connections with those that share their views. However, within the UK community, despite the polarisation in the climate change debate, data also indicated strong communications between people with opposing opinions about climate change (Pearce et al., 2014), suggesting the possibility of potentially building greater mutual understanding between groups with differing beliefs.

Social media has also previously been used to examine public perceptions of climate change (Segerberg and Bennett, 2011; Auer et al., 2014; Kirilenko and Stepchenkova, 2014; Pearce et al., 2014, 2019). Satchwell (2013) explored children’s understanding of climate change using Twitter conversations as an observational method, concluding that access to information about climate change does not necessarily translate to actions taken for climate change mitigation. Kirilenko and Stepchenkova (2014) collected a total of 1.8 million tweets in 2012 and 2013 to assess people’s perceptions and impressions of climate change, depending on their spatial location and potential exposure to news or events related to climate change. Kirilenko and Stepchenkova (2014) found that the climate change discourse in Twitter showed high temporal variability and intensified significantly with major new events, such as Hurricane Sandy that impacted the Atlantic in 2012.

Veltri and Atanasova (2017) introduced the use of semantic analysis and natural language processing (NLP) tools for the analysis of Twitter data and climate change. The researchers’ findings suggested that the most salient topics expressed along with “climate change” were “awareness,” “flood,” “action,” and “energy.” The researchers also found four main thematic clusters emerging from the discourse around climate change: (i) calls for action and awareness, (ii) causes and consequences, (iii) policy debates, and (iv) local events associated with climate change. These findings develop a broad understanding of public opinions of climate change by identifying the formation and evolution of themes and their frequency in the discourse of climate change. However, Veltri and Atanasova (2017) did not delve further into the nature of the discourse within each of the thematic clusters identified to consider, for example, the types of action called for. Indeed, there is no current evidence, of which the authors are aware, of the assessment of Twitter users’ discourse around the actions for climate change mitigation, or of how these may have changed over time during the global pandemic.

This paper reports the results from a longitudinal study, examining public perceptions of actions for climate change mitigation through sets of tweets streamed at three different points in time during the COVID-19 pandemic, contributing to the understanding of the publics’ perceptions of actions taken to mitigate climate change and the changes in these perceptions over time. We used Twitter data collected in February (time 1), June (time 2), and October (time 3) 2020, respectively. We hypothesised that the global pandemic may impact the frequency with which actions for climate change mitigation is mentioned within tweets. We also predicted that the topics emerging within tweets relating to actions for climate change mitigation may change over time, differing both in terms of the frequency with which they emerge and the nature of the topics.

## Materials and Methods

### Sampling Procedure

Latent Dirichlet Allocation (LDA) topic modelling is an NLP technique described as an unsupervised machine learning technique that analyses text data to determine clusters of words or topics for a set of documents. In the case of social media, topic models are used to analyse public reactions and conversations that happen online between people by extracting and identifying patterns in the popular topics shared on platforms such as Twitter (Sun et al., 2017; Xu et al., 2017). By topics, we refer to the collection of dominant topic words that often work as representatives; the topic words can help us define the main topics of discussion. In the context of text modelling, the topic probabilities provide an explicit representation of the content of the documents (Blei et al., 2003). The researchers decided to use the LDA model based on previous literature using this method (Ostrowski, 2015; Chen et al., 2016) and also due to its unsupervised classification features, not requiring for predefined topics, was crucial to the evaluation of hidden patterns or topics within the discussions of action tweets for climate change mitigation.

We used R software to stream unique tweets written in English with the Rtweet package at three different points in time with the keywords “climate change,” “sustainable,” “environmental + action,” “be green,” “global warming,” and “save the planet.” We streamed all the available unique tweets with the relevant keywords daily, and then compared them with tweets collected on previous days and removed duplicates; we kept on doing this process until we reached a minimum of 300,000 tweets streamed at each time point. On the first stream, time 1, we obtained 318,749 tweets (4th–13th of February 2020); time 2, 305,851 tweets (17th June–2nd of July 2020); and time 3, 303,636 (12th–23rd of October 2020). A total of 928,236 tweets were streamed across the three points in time; all tweets were anonymised prior to any further analysis. The rationale behind the choosing of these time periods was based on the stages of the pandemic in the UK; the first set of tweets streamed on February (time 1) was collected to gather data on the early stages of the pandemic, the second set of tweets (time 2) was collected after designing the study (4 months later) to gather data just after the first lockdown in the UK, and time 3 was collected 4 months after time 2 just as the UK was reconsidering their actions towards the second wave of the pandemic in the UK. Thus, the gaps between the streams also allowed an evaluation of the perception of climate change mitigation actions during the different stages of the pandemic. For tweets classification and analysis, we used Python.

Since we were interested in actions for climate change mitigation, we selected “action” tweets from the tweets dataset at each time point. In order to identify the “action” tweets, one researcher independently classified a total of 3,949 tweets from the first data set (Pitsilis et al., 2018; Ljevar et al., in press). A second rater then classified a random sample of 750 tweets from the same sample of tweets using criteria identified by the first researcher to discuss and validate the tweet’s classification. The inter-rater reliability Cohen’s kappa test showed a good level of agreement between the raters (2 raters), κ = 0.64 (95% CCI, 0.588–0.700) *p* ≤ 0.05.

An “action” tweet was considered as any tweet that would describe a behaviour aiming to mitigate climate change; see Table 1. For example, a tweet was considered an “action” tweet if it identified the actions that others ought to do to mitigate climate change (e.g., “*…If you lecture about climate change, sell your private jets, use mass transit and bike or walk; when possible, go vegan…*”); reported the start of a particular behaviour intended to mitigate climate change, in either in individual or collective form (e.g., “*Jane Fonda recycled a 2014 gown …to make a statement about climate change*,” or “*From banning pesticides to growing green roofs on bus stops … communities are trying to save the bees*”). Action tweets also included the development of policies to tackle climate change (e.g., “*The government has announced funding to support 13 projects that will plant 50,000 trees in total across England to support the fight against climate change*”); pointed out others’ perceptions of environmental actions that, in their opinion, do not help to mitigate climate change (e.g., “*…Being vegan will not save the planet…*”); and highlighted or condemned other people’s actions around climate change (e.g., “*You are using a lithium battery to operate your phone climate change, HYPOCRITE*”).

**Table 1:** Description of the types of actions considered for the classification of “action tweets.”

Type of action	Description	Tweet Examples
The initiation of a behaviour	Reporting the start of a particular behaviour with the intentions to mitigate climate change. This can either be individual or collective.	“Jane Fonda recycled a 2014 gown at the Oscars Sunday night to make a statement about climate change” “From banning pesticides to growing green roofs on bus stops here are 5 ways communities are trying to save the bees”
Behaviour to adopt for climate change mitigation	Identifying the actions that others ought to do to mitigate climate change.	“If you lecture about climate change sell your private jets use mass transit and bike or walk when possible go vegan etc. …” “Does Eating Less Meat Fight Climate Change CA Study Says Yes” “Let’s end capitalism and save the planet Green revolution”
Behaviour that should be stopped to tackle climate change	Identifying behaviour that contribute to climate change and a call to individuals to stop this behaviour.	“Our attitudes to flying need to change before its too late” “Southeastern trains please turn off the heating on your unbearable trains Thought your supposed to be green Its not free energy”
Monetary options for climate change mitigation	Investmenton initiatives focused on tackling climate change.	“Amazon founder Jeff Bezos commits 10bn to fight climate change”
Political actions for climate change mitigation	Calling on leaders and heads of organisations to act to mitigate climate change.	“Sign and send the petition Urge your governor and state legislators to urge state pension fund divestment from fossil fuels We cannot continue to fund climate change Write one here”
Government actions for climate change mitigation	Development of policies devoted to a particular action to tackle climate change.	“Netherlands 515 billion pension fund to accelerate cuts to fossil fuel investments Article AMP Reuters” “…Bipartisan Climate legislation to put a Price On Pollution is key to addressing climate change…” “The government has announced funding to support 13 projects that will plant 50,000 trees in total across England to support the fight against climate change.
Actions that do not help climate change mitigation	Users’ perceptions about environmental actions that do not help to mitigate climate change.	“So to save the planet with electric cars and mars bases Elon is chopping down 225 acres of forest for his new European factory and chucking tons of carbon into the atmosphere for escape velocity Sounds like a plan” “Being vegan will not save the planet …”
Goals on climate change mitigation	No clear actions or behaviour reported but describe a desired end goal (reducing carbon footprint).	“For years now Pacific Island governments have been pleading with more developed countries to phase out the use of fossil fuels and rapidly transition to cleaner sources of energy”

**Figure 1:**
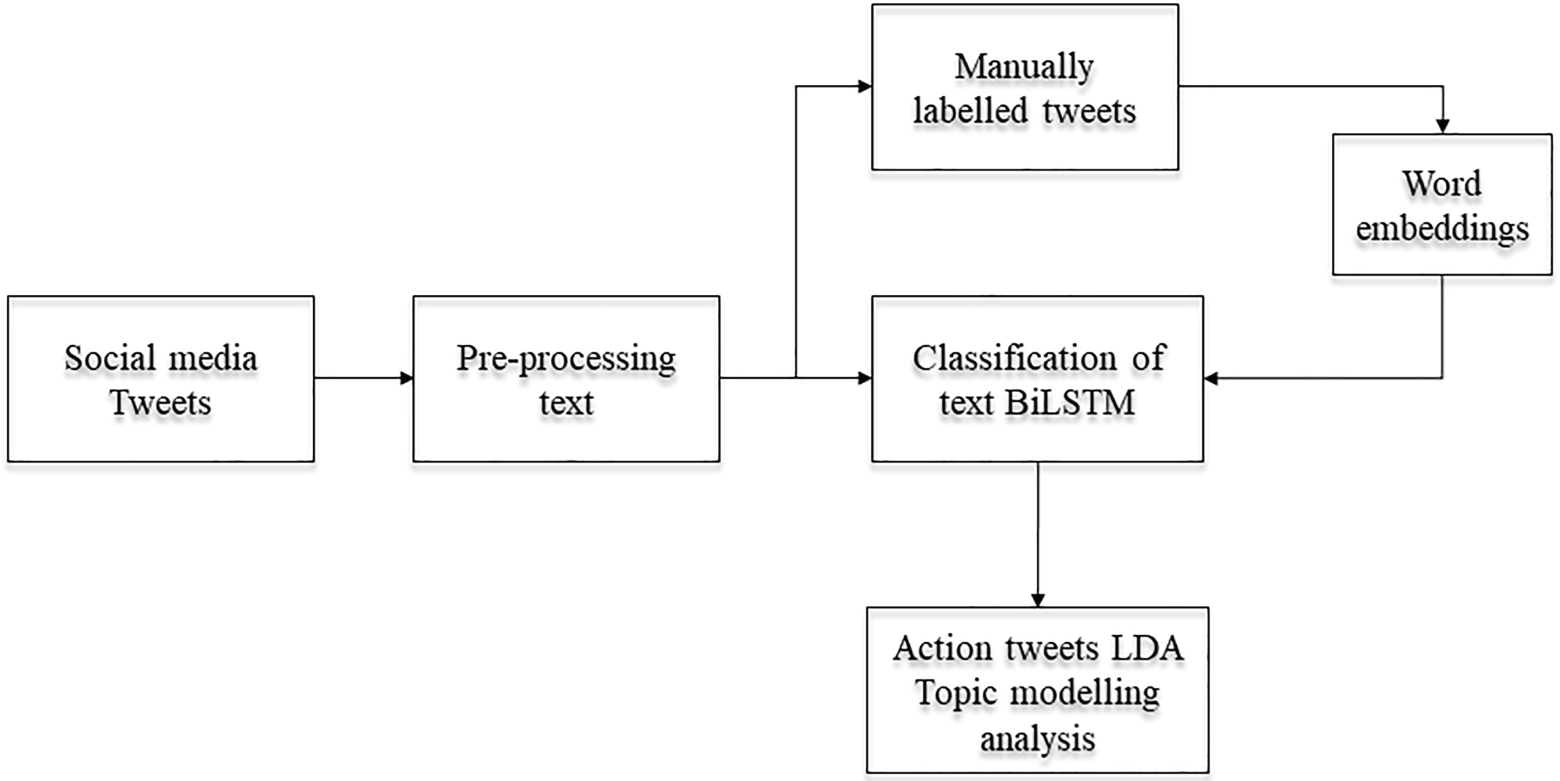
Diagram of tweets processing and analysis. Tweets were streamed from social media on the three-timepoints, then pass through a pre-processing phase (removal of punctuation, mentions, etc.). A Bi-Directional Long Term Memory (BiLSTM) model was used for the clarification of tweets into action and non-action tweets. The inputs for the model were the word embeddings created from the manually labelled tweets. The action tweets detected from BiLSTM were then used for the analysis of the content with unsupervised technique LDA Topic Modelling.

Detecting “action tweets” was considered a classification task with a two-class variable. Data were split into training (80%) and test (20%) sets. We used neural network architecture in this task, as it has previously been shown to be successful when trained in language properties (Cocos et al., 2017). A bi-directional long-term memory (BiLSTM) was used to classify climate change tweets as “action” or “nonaction” tweets. Recent literature has shown that neural network models, such as BiLSTM, perform better in cases beyond sequence prediction problems and in the field of text classification (Cocos et al., 2017; Ljevar et al., in press). BiLSTM transforms words in sequences of vectors to process them across the different documents and examine the temporal dependencies of words within the data. Thus, the input features for the BiLSTM model were word-embedded vectors that were created from the manually labelled Twitter data (see Figure 1) Performances were then tested on the remaining set of tweets, and the quality of performance was assessed using the classification prediction. Prediction accuracy of 88.3% was obtained, along with satisfactory recall (0.74) and precision (0.93) scores. This classification prediction was deemed as successful, and the model was used on all three sets of tweets to automatically classify the tweets into “action” and “nonaction.” As a result, a total of 165,872 “action” tweets was obtained across the three time points of datasets.

### Procedure Unsupervised Machine Learning Analysis

For the identification of the topics for each set of tweets, we selected the “action” tweets only, since we were interested in public perceptions of actions for climate change mitigation. For this task, unsupervised machine learning was used, specifically, the Latent Dirichlet Allocation (LDA) model, with the Gensim package from the programming software Python. LDA is an unsupervised machine learning technique, indicating that there is no predefined list of tags. Topic modelling scans a dataset of text, detecting words and phrase patterns within the text and clusters the word groups and similar expressions that characterise the dataset. We analysed each set of tweets separately to identify relevant topics discussed for each of the three datasets of tweets.

After analysing the text content of all the tweets within one dataset, LDA calculates the percentage of each topic present within each set of the tweets. The output of the LDA model thus lists all the topics, along with the frequency of how often a topic was used within the text of the tweets. It is important to highlight that there can be more than one topic within a tweet.

For all three data sets of action tweets, we filtered out stop words (e.g., “*a*” and “*the*”) and the keywords that were used to stream the tweets (e.g., “*climate change*” and “*save the planet*”). We then lemmatised the remaining words; this process transforms the words into their roots (e.g., *walking to walk*), and then created a dictionary from the lemmatised data and converted this to a bag of words corpus; this was the main input for the LDA model. Then, we identified the optimal number of topics that best fitted the data by running many simulations of the model and adjusting the number of topics in order to find the number that optimised the objective metric; in this case, the objective metric was the coherence score (Kapadia, 2019). For topic optimisation, we used a coherence score rather than a perplexity score sometimes used, since it has been shown to correlate better with human interpretability (Kapadia, 2019). The number of topics that held a coherence score of 0.54 for time 1 was 9 topics; for time 2, we obtained a model with 12 topics and a coherence score of 0.55; and for time 3, we obtained a model with 11 topics and a coherence score of 0.5. After setting the number of topics for each set of “action” tweets, we assessed the differences in the Twitter data between time points in terms of number of topics, frequency, nature of topics, relevant topic words, and the emergence of new topics. Table 2 provides an overview of the process of tweets collection and the final number of action tweets used in the topic modelling analysis.

**Table 2:** Overview of total tweets, “action” tweets, and topic modelling analyses.

Times	Total of tweets streamed	# of action tweets	# of Topics	Coherence Score
Time 1 (February 2020)	318,749	33,659	9	0.54
Time 2 (June 2020)	305,851	72,798	12	0.55
Time 3 (October 2020)	303,636	59,415	11	0.50

Number of total tweets streamed for each time-point along with the action tweets identified by the BiLSTM model for each of the three sets of tweets. Coherence scores were obtained from topic modelling analysis of the number of action tweets reported to be acceptable at levels ≥0.50.

In order to define the outcomes from the topic modelling analysis, we will develop topics from the clusters of words that the model defines (henceforth referred to as “topic words”), along with the percentage of tweets that relate to each topic (Table 3). The interpretation of the topics was carried out by evaluating the collection of dominant topic words within each topic; this was supported with the manual examination of tweets to understand the context where the topic words were used. Two researchers oversaw the assessment of the collection of dominant topic words for each topic to define all topics in the three datasets.

**Table 3:** Salience of topics for each set of tweets.

Topic	% of data Time 1	% of data Time 2	% of data Time 3
Government actions	52.9	21.6	2.8
Environmental behaviours	33.6	46.9	69.9
Awareness	3.7	2.3	1.3
Collective actions	3	1.4	1.4
Organisation’s actions	1.8	0.8	1.5
Protest to government	1.5	1.5	-
Social Network initiations	1.3	-	-
Awareness and Fairness	1.2	-	-
Third party actions	1	1.6	1.3
Eco-friendly products	-	11.8	10.2
Food	-	7.3	-
Nature conservation	-	2.9	-
Sustainable production	-	1.1	3.6
Innovative solutions	-	0.8	1.8
Support farmers	-	-	4.4
Consequences of climate change solutions	-	-	1.8

## Results and Discussion

The following data show the results of the topic modelling analyses of the “action” tweets streamed at the three different points in time during the COVID-19 pandemic (February, June, and October 2020). Table 2 shows the total number of “action” tweets identified for all three sets of data. Findings show an increase of more than double the number of action tweets found at time 1 (33,659), for time 2 (72,798), and similar high amounts at time 3 (59,415). Table 4 shows the brief statistics of the total number of words, the number of unique words, and the average of relevant words per tweet for the “action” tweets.

**Table 4:** Brief statistics of words for action tweets.

Time	Total number of words	Unique words	Average words per tweet
Time 1	225,503	24,365	6.7
Time 2	606,128	36,659	8.3
Time 3	536,703	29,564	9

The number of words was calculated after the removal of stop words (e.g., “a,” “the,” “and”), and keywords used for streamed tweets, considering only the words that were used for the topic modelling analysis.

Overall, the most salient topic words across the three sets of tweets were “energy,” “food,” “people,” and “help.” The topic words “tax,” “money,” and “funding” were observed at time 1 and then became less frequent within the time 2 and time 3 datasets. Among the most frequent topic words observed in time 1 tweets were “carbon,” “money,” “energy,” “tax,” “fuel,” “customer,” “fund,” “fossil,” “emission,” “trade,” “coal,” “address,” and “clean.” For time 2, the most frequent topic words were “energy,” “food,” “development,” “future,” “production,” “waste,” “farmer,” “recovery,” “people,” “solution,” and “agriculture.” And for time 3, the most frequent words were “energy,” “food,” “future,” “development,” “people,” “industry,” “farmer,” “plastic,” “fashion,” and “packaging.” The topic words “farmer,” “plastic,” “food,” “packaging,” “waste,” and “agriculture” seemed to be more frequent across the tweets from time 2 and time 3, but not in time 1.

The analyses of the topic modelling included the interpretation of the topics found by the model. In order to interpret these topics, we examined the most relevant topic words for each topic, detected sections of tweets that presented these topic words, and used these as the basis for naming the topics for each of the three time points. Table 3 compares the frequency of topics identified in datasets between time points.

While tweets on climate change mitigation are predominantly focused on the topic of “*government actions*” in time 1 (52.9%), we found its relevance decreased for time 2 (21.6%) and time 3 (2.8%). The drop in relevance of the topic of “*government actions*” for time 2 and time 3 could be due to governments’ actions focusing primarily on the pandemic at times 2 and 3, resulting in users talking more about the government and COVID-19 than the government and climate change. Tables 5–7 provide further information on each topic identified, along with examples of tweets that mentioned the topic. By comparing the topics that were present at different time points, we can see that some topics, labelled as the same, differ with regard to the relevant topic words within the topic. For example, the topic of “*government actions*” in time 1 was represented with the topic words “money,” “fund,” “fuel,” “fossil,” “tax,” and “investment,” whereas for, time 2, representative topic words for this topic were “development,” “recovery,” “COVID,” “resilient,” and “report”; and for time 3, representative topic words for the same topic were “plan,” “healthcare,” “act,” “insurance,” “data,” “police,” “socially,” and “council.” These results suggest that the discussions encompassed by the topic of “*government actions*” differed over time. One possible explanation is that time 1 could have been influenced by the early voting for presidential elections in the US, occurring in February 2020; we considered that this event may have politicised the tweets relating to climate change mitigation at time 1, driving comments around climate change mitigation to be focussed more on the candidates and not on the problem itself.

**Table 5:** Time 1. Labelled topics and topic words within tweets from February 2020.

Topic’s label	Description	Examples of tweets	Number of Tweets and % of data	Topic words
T1. Government Actions	Government actions to mitigate climate change, from funding initiatives to imposing taxes.	“…A carbon tax can incentivise corporations eg UKs Climate Change…” “…Our current national policy is to spend billions in taxpayer money increasing climate risk.”; “…A Republican plan to address climate change fails to address carbon pollution…”	17,803 (52.9%)	Carbon, emission, money, fuel, fund, fossil, tax, address, company, way, government, people, industry, dollar, taxis, oil, investment.
T2. Environmental behaviours	Observing the different industries or types of behaviour that may help with climate change mitigation.	“…mistakenly believe that forest bioenergy and long lived wood products are solutions…” “…providing eco energy product solution to leading a more sustainable lifestyle”;”… you can cut the risk to yourself and the planet by eating more plant based foods like this spicy jackfruit crispy taco…” “…Investing in agriculture can address not only hunger and malnutrition but also other challenges including poverty climate change and unsustainable production and consumption…”	11,309 (33.6%)	Energy, people, food, renewable, car, clean, coal, tree, sustainable, waste, air, power, solar
T3. Awareness	Promoting awareness about climate change around international events.	“… Prize in Food and Agriculture Sciences … and aims to improve crop production and hardiness helping to address challenges of global population growth and climate change…”; “…Awards…support in biodiversity conservation fisheries management combating wildlife trafficking and climate change mitigation….”	1,246 (3.7%)	Award, liberal, trade, mention, direct, third, essential, diversity (div), pocket, video, great, elite, evil, reusable, know, pension, airport.
T4. Collective actions	Collective behaviour, noting behaviour undertaken, or that could be undertaken, as part of a community or a group to combat climate change.	“…Imagine how much we could reduce that if there was public demand for alternative milk protein sources…”;”… We can save Earth Its going to take collective action from big companies small companies nation states global organisations and individuals…”; “From banning pesticides to growing green roofs on bus stops here are 5 ways communities are trying to save the bees…”	1,011 (3%)	City, ready, milk, bee, collective, combat, customer, capital, open, burn, teacher, baby, network, corrupt, mobility, college, ways_communities
T5. Organisation’s actions	Describing what organisations are doing to combat climate change.	“…Airlines may need to reduce passengers to take off due to climate change…”; “changing aircraft altitude could cut flights climate impact in half…”; “…how going digital and eradicating paper receipts sets companies on a sustainable path in the green economy…”	605 (1.8%)	Wildlife, decade, skill, degree, reduce_passenger, scientist, block, extraction, takeoff_due, aircraft, ditch, greeneconomy, illegal,
T6. Protests to governments	Protesting to authorities about climate change.	“Just like us Jane Fonda who flies to DC weekly to get arrested protesting climate change…”; “…hear about an Extinction Rebellion protest to try and get Tom DiNapoli to divest from fossil fuels from and questions from town hall Sat about impeachment climate change…”	504 (1.5%)	Town, run, bullshit, saving, gold, hypocrisy, dc_weekly, metal, show, radical, toward_solving, change trump,
T7. Social network initiatives	A call to the public to show support for climate change campaigns.	“…Tomorrow is one more chance to catch them this season We need your photos to create a record of changes to our coast to raise awareness about climate change and to help California plan…”; “Thurs Feb 6 is SweaterDay This is an opportunity to raise awareness about climate change energy conservation and show how together we can be a part of the solution…”	438 (1.3%)	Tomorrow, platform, generate, photo, actor, show, solid, burning, sweater, positively, thermostat, generate_value, laundry,
T8. Awareness and fairness	Expressions of inconsistency within other people’s actions to mitigate climate change	“…Oscar nominees get a 225,000 gift bag arrive in private jets and limos but they want to lecture you on income inequality and climate change….”; “Like lecturing on climate change while having multiple homes private jets helicopters Private armed security you think rules dont apply to U”; “…explain how you said youre fighting for climate change but then you fly on a personal jet Then you have the nerve to try to hide behind your staff when you were spotted…”	405 (1.2%)	Twitter, crazy, hot, decent, push, find, bicycle, technew, helicopter, limo, tv, equity, hide_behind.
T9. Third party actions	Observing what third parties and public figures do about climate change mitigation.	“David Gilmour sold his guitars for 215 million and donated everything to fight climate change…”; “Orsted one of the worlds biggest developers of offshore wind farms wants to decarbonize heavy industries using Hydrogen to achieve climate change goals….”	338 (1%)	Hydrogen, climateactnow_auspol, global warming, utility, visit, guitar, court, persuade, art, mansion, flight_shaming, mansion, utility.

The description column is based on the interpretation of the topic based on the relevant terms and analysis of the tweets for further context. An example of tweets shows fragments of the tweets where the topic was present.

**Table 6:** Time 2. Labelled topics and topic words within tweets from June 2020.

Topic’s label	Description	Examples of tweets	Number of Tweets and % of data	Topic words
T1. Environmental behaviour	Observing the different industries or types of behaviour that may help with climate change mitigation.	“…Transitioning away from coal to sustainable biomass is essential in the fight against climate change…”; “… the focus will be on a green future investing in sustainable transport the focus will be on a green future investing in sustainable transport…”; “….we need is to stop buying new cars share the ones we have walk and cycle work …”	34,142 (46.9%)	Way, energy, people, year, emission, money, time, company, new, carbon, industry, infrastructure, solution., transport, car, plan, community
T2. Government actions	Government actions to mitigate climate change.	“…Vote now for D2T2 project and help promote ocean energy in EUs largest sustainable energy event…”; “offers world governments a Sustainable Recovery Plan to create millions of jobs and put emissions into structural decline Healthy Recovery BuildBackBetter…”;“EU is revisiting its transport vision for the next decade a Sustainable and Smart Mobility Strategy…”	15,724 (21.6%)	Development, recovery, investment, future, economy, fund, technology, innovation, covid, resilient, growth, policy, government, energy, report.
T3. Eco-friendly products	Use of eco-friendly products, brands, and other resources to reduce impact of climate change.	“Its super frustrating that sustainably and environmentally friendly products are so hard to find theres hundreds of …ones wrapped in pounds of plastic…”; “We installed Solar WaterHeating and SolarPV systems at ….a luxury and ecofriendly resort”; “This brands latest collection named remade is repurposing their own fabric waste to create a new line of clothing”	8,590 (11.8%)	Plastic, product, eco-friendly, clothing, waste, fashion, help, shop, alternative, nuclear, chemical, ethical, paper, reusable, pandemic, biodegradable, recycle, brand
T4. Food	Alternatives in food industry to mitigate climate change	“…Food production is the biggest contributor to climate change but one third of food is wasted We want to change that…”; “…Sustainable naturally raised meat production integrated closely with plant production is the way to go it is not even debatable…”; “…Canadian meat dairy are my countrys sustainable organic natural food sources which my ancestors survived from for hundreds of years Ur not changing that…”	5,314 (7.3%)	Food, farmer, farm, agriculture, meat, fish, diet, animal, healthy, sea, production, crop, organic, dairy, soil, water, nutrition.
T5. Nature Conservation	Conservation of green areas and species	“Well done Great to see the FMCG giant helping to fund reforestation water preservation and carbon sequestration in a commitment to cut company emissions”; “…Check how retailers are doing in sourcing responsible deforestation free palm oil by using the PalmOilScoreCard…”; “…Nuclear Power Plants as they can Generate Supply Affordable Reliable Sustainable 247 Power at Affordable Costs also keep in Mind that Coal Oil Gas are NOT Sustainable globally”	2,111 (2.9%)	Forest, black, electric, present, necessary, plant, conservation, species, loan, driver, climatecrisis, reliable, control, village, secondary, nuclear_power, vulnerable, global, stop, heat
T6. Awareness	Promoting awareness about climate change	“Roadhouse management takes responsibility and the initiative to be more socially and environmentally conscious actively engaged in creating sustainable and a quality road for our clients”; “…environmentally conscious teen …who believes we can all continue to help the environment and remain sustainable even when we are stuck at home…”; “…We have 500 innovations just around sustainable packaging and plastic pollution…”	1,674 (2.3%)	Packaging, bag, fair, range, app, person, env_conscious, aid, man, collectively, consequences, review, incentive, labour, stable.
T7. Third party actions	Observing what third parties and public figures do about climate change mitigation.	“bro arent you using cow milk for your ice cream cows are responsible for climate change as well…”; “a project that combines chitosan from fungi with protein extracted from corn and milk waste to create a new sustainable type of fabric”; “The sustainable decision from the beloved Arab designer comes at a time when we all need to step up for mother nature and reduce our environmental impact”	1,164 (1.6%)	Massive, milk, mining, designer, scientist, metal, furniture, police, racism, carbon_neutral, biomass, river, transfer, gold, cocoa, film, non-profit
T8. Protest to governments	Protesting to authorities about climate change.	“…Leaving the European Union now means Massive increases of UK Carbon Footprint and Contribution to Global Warming due to increase Transport of goods Racist English…”;” Some are already seeing COVID19â€™s stimulus packages as an opportunity to spur green growth The United Kingdom plans to spend 250 million pounds of its stimulus package on walking and cycling infrastructure…”	1,093 (1.5%)	Economically, phase, cycling, timber, working, outdoor, textile, vote, colleague, glass, exchange, bet, wool, action_climate, auspol, greenhouse, evolution, summit, minute.
T9. Collective actions	Collective behaviours, listing behaviours to be undertaken as part of a community or a group to combat climate change.	“Newly Guaranteed Fair Trade Enterprise Machakos Cooperative Union ensures that sustainable livelihoods for producers means putting environmental focus on their production and farming”; “…celebrates 26 years of unique hospitality…. the trendsetter in sustainable development and the pioneer of eco initiatives celebrates 26 years of breaking barriers and lighting the path for a sustainable hospitality industry”;” globalists climate alarmists etc. dont want electricity available to the masses They know electric cars solar panels and windmills are not sustainable So their solution is communism and energy poverty”	1,019 (1.4%)	Threat, regulation, boost, category, hospital, adapt, cooperative, electric_car, corona, innovate, hospitality.
T10 Sustainable production	Alternative sustainable products and actions to mitigate climate change	“Over the next three years in The Netherlands will be investing 55 million in a testing facility for the development of sustainable plastics…”; “…replacing palm oil with another oil will just mean more deforestation Choose sustainable palm oil and support local initiatives…”; “Paying farmers a living wage is essential to ensuring sustainable coffee production…”	801 (1.1%)	Coffee, palm_oil, death, recycled, Indian, hotel, chocolate, manufacture, rainforest
T11 Organisation’s actions	Describing organisation’s actions to combat climate change.	“FYI systemic barriers to energy efficiency low zero carbon world banks funding of high carbon polluting investments”; “Amazon pledges 2bn investment to fight climate change…”; “EU Parliament adopts measure to boost green investment The European Parliament has adopted a key piece of legislation to add to the European Green Deal whose aim is to increase private sector investment in sustainable and eco-friendly projets”	584 (0.8%)	Bank, powerful, museum, disease, trump_admin, amazon_pledge, parliament_adopt, registration, crash, fire.
T12 Innovative Solutions	Promotion of innovative initiatives for tackling climate change	“desserto a highly sustainable plant based vegan leather made from cactus”; “…Australia aims to produce vehicles that are sustainable in every aspect made from strong biocomposite body materials that will maximise energy efficiency with a hybrid power system…”; “As the pandemic forces us to rethink our old approaches and ways we call on our industries businesses policymakers and households to promote sustainable gastronomy by respecting food and everything that goes with it…”	582 (0.8%)	Aim, park, lesson, leather, behaviour, dangerous, press, composite, telehealth, fiberboard, paint, gastronomy.

The description column is based on the interpretation of the topic based on the relevant terms and analysis of the tweets for further context. An example of tweets shows fragments of the tweets where the topic was present.

**Table 7:** Time 3. Labelled topics and topic words within tweets from October 2020.

Topic’s name	Description	Examples of tweets	Number of Tweets and % of data	Topic words
T1. Environmental behaviour	Observing the different industries or types of behaviour that may help with climate change mitigation.	“So you want to burn all the fossil fuels on the planet accelerating global warming and at the final years when Fossil fuels are all extracted and burn have a massive crises cus you ran out of energy probably ending society as he know it…”; “Hydropower developers conservationists and former energy officials announced a major collaboration yesterday to burnish hydros credibility as a renewable resource and expand its role in fighting climate change”; “…FoodHeros we reiterate our commitment to contribute to creating a robust sustainable resilient food system…”; “…Irish dairy farming which has been found to be highly sustainable with regard to water Almond farming in California uses tens of billions of water per annum There is no comparison”	41,531 (69.9%)	Energy, food, development, future, way, new, solution, people, system, industry, community, emission, company
T2. Eco-friendly products	Use of eco-friendly products and brands and other resources to reduce impact of climate change.	“…the largest organic vegetable producer in the USA is exhibiting new products and sustainable packaging initiatives this week at the first ever virtual Produce CalOrganic…”; “…just launched ReStyle 2020 a fashion collection upcycling discarded materials from automotive manufacturing and scrapping into fashionable products…”; “…fashion brand will be the FIRST 100 biodegradable sustainable and eco friendly fashion brand And its created by a black woman”;	6,060 (10.2%)	Product, packaging, brand, material, fashion, cheap, full, eco-friendly, plastic, clothing, ethical, collection, luxury, slow, lockdown, sustainably.
T3. Support Farmers	Promote awareness about the future of Common Agricultural Policy in Europe and promoting to support farmers.	“empower farmers to provide sustainable food for our communities Dont get fooled and vote for a future of CAP…”; “the future of sustainable farming in Europe is at stake after in the struck a deal that will syphon tens of billions of euros to big farmers with few environmental conditions FutureofCAP”; “Heres a funding opportunity for female entrepreneurs building a sustainable future…”	2,614 (4.4%)	Pay, matter, manager, true, person, entrepreneur, common, wood, art, European, delivery, investing, futureofcap, disaster, fix, ecological, corporate, pesticide.
T4. Sustainable production.	Alternatives on sustainable production to mitigate climate change	“…Various clothing stores are specialized for providing the ethical and sustainable apparels to its customers…”; “Useful info to share from when youre dairy free get challenged about the source of the soya you use I only buy from EUCanada sustainable sources dairy free environmental”; “…The demonstration farm in Dowth anmhi will develop this sustainable farm platform which can underpin irishbioeconomy development buildbackbetter”	2,139 (3.6%)	Store, retailer, ban, gift, dairy, engender, exciting, legislation, products_sold, bottle, loan, beef, trading, locally, heat, border
T5. Government actions	Government actions to mitigate climate change.	“Climate change is real We need to stop the spread of covid19 help families in need make education healthcare free or affordable”; “…whether we believe in climate change and accept a womans healthcare is not the governments business to regulate I also want us to accept healthcare is a human right”; “He would build infrastructure fight climate change raise wages guarantee health insurance coverage expand childcare…”; “There is no insurance policy Governments can buy against the catastrophe of abrupt climate change … We must invest in solar radiation management infrastructure to balance Earths energy now along with CO2 drawdown…”; “…electric motorcycle taxis and recycling 500 billion bottles for a sustainable Thailand a UN Resident Coordinator…”	1,664 (2.8%)	Plan, healthcare, act, insurance, data, expertise, police, socially, council, taxis, stability,
T6. Innovative solutions	Promotion of innovative initiatives for tackling climate change	“Want to fight climate change I just signed up for first wooden debit card powered by …80 of profits to responsible reforestation Sign up now and theyll plant 3 trees plantchange woodendebitcard”; “Solar Powered Luxury Yachting by SILENTYACHTS Yachting SILENTYACHTS create an independent and sustainable yachting experience…”;” Great to see supermarket chain Tesco furthering its mission to eliminate deforestation from their own supply chain”	1,070 (1.8%)	Profit, powered, sign, they’ll_plant woodendebitcard, tree_plantcharge, democracy, taxpayer, reserve, faster, wide, supermarket, provision, universal, sailing_boat
T7. Consequences of climate change solutions	Paying attention to the consequences of climate change mitigation strategies and other events.	“Electric cars are to climate change what electric cigarettes are to lung cancer”; “And this is an ever reducing problem what with emissions control electric cars but the intensified pressure on local ecosystems by urban centres and the non sustainable agriculture models used to feed them is getting worst…”; “…FDA of the United States of America Stop Using Sharks in COVID19 vaccine Use EXISTING Sustainable Options Sign the Petition…”;” Legacy cities Detroit Gary Cleveland Baltimore etc. should be allowed to establish their own utility companies based on wind and solar energy that belongs to everyone It would solve for climate change and economic racial justice at the same time …”	1,069 (1.8%)	Mining, interest, director, foundation, make, electric_car, Australian, vaccine, pool, film, renewable, accelerate, beach, please, fake, virus, lithium, solar_energy
T8. Organisation’s actions	Describing organisation’s actions to combat climate change.	“Antarctica shouldnt strive for cost effective sustainable energy without worry”;” Alaska Airlines and Microsoft sign partnership to reduce carbon emissions with flights”;” Beer company… in Colombia introducing an Airbnb style platform showcasing Colombian ecohotels that…sustainable tourism…”	891 (1.5%)	Forever, cost_effective, fabric, rapid, metal, hotel, airline, audience, conventional, innovator
T9. Collective actions	Collective behaviour, noting behaviour undertaken, or that could be undertaken, as part of a community or a group to combat climate change	“…climate neutral farms and food businesses reducing dependence on chemical inputs reducing waste and sustainable diets Projects will fundamentally depend on cross organisational…”;”… Elements of capitalism socialism can be used to drive an economy with social ecological sustainable economic goals a balanced approach”; “Sustainable behavior begins at home kick starts the International EwasteDay caign by pledging to dispose off old electronics responsibly…”; “pull nitrogen out of the air and fix it into a form that other plants can use… These and other insights can be used to design and create sustainable agricultural systems… What is Permaculture”; “…the 5 year sustainable development bond raised 800 m British pounds will finance AIIB in sustainable infrastructure unlocking new capital technologies ways to address ClimateChange”	829 (1.4%)	Chemical, bag, bond, solid, wealthy, attention, cancer, rain, comfort, consume, tea, ewaste, evolution, socialism, nitrogen.
T10 Awareness	Promoting awareness about climate change.	“Continually impressed with the ingenuity of Utah companies a STEP Grant recipient created a sustainable way to make jeans from coffee grounds and plastic water bottle…”; “Young people all over the world are working hard to raise awareness about ClimateChange …Climate change is real and we need to dramatically reduce greenhouse gas emissions”	775 (1.3%)	Coffee, cover, minimum, realistic_source, code, river, eat, trash, raise_awareness, fundraising, income
T11 Third party actions	Observing what third parties and public figures do about climate change mitigation.	“…An organism commonly used in biology labs engineered to take carbon out of the air … Synthetic biology could help mitigate climate change”; “Apple has a strategy to encourage customers to purchase almost annually for a new iphone…Environmentally this is not a sustainable product strategy …”; “We applaud <U+0001F44F> Apple’s effort to reduce ewaste emissions by NOT including a USBC charger with iPhone 12…”; “Instead of focusing on what travelers shouldnt do or limiting behaviors highlight instead deeper more unique experiences that sustainable tourism provides…”; “A big step for the airport … added 5 new electric buses which will result in a reduction of 50,000 gallons of diesel fuel each year …”	773 (1.3%)	Engine, destruction, inclusion, island, integration, diesel, disease, trial, planting, tv, veggie, territory, soy, destination, plate, apple, petroleum, tv, intelligence, edge, traveler, applevent.

The description column is based on the interpretation of the topic based on the relevant terms and analysis of the tweets for further context. An example of tweets shows fragments of the tweets where the topic was present.

The “*environmental behaviour*” topic commenting on actions for the mitigation of climate change seemed to be the most prominent topic for time 2 (46.9%) and time 3 (69.9%), in comparison to time 1 (33.6%). One plausible reason for the increase in the relevance of the topic is that people may have started to reflect on their behaviour over the course of the pandemic that was incidentally more pro-environment due to restrictions, perhaps partly due to increased time that some had available for this. Indeed, research indicates that individuals reflecting on their actions towards climate change and the implications of climate change on their lives may have an impact on their behavioural intentions for climate change mitigation (Rickard et al., 2014). Another explanation of why the topic “*environmental behaviour*” had an increase in the amount it was mentioned may be due to the increase in non-environmental behaviour individuals had to undertake due to COVID-19 (e.g., wearing face masks and using disposables and hand gel) having a compensatory effect on other environmental behaviour (Hope et al., 2018; Capstick et al., 2019). So, individuals may have mentioned actions to mitigate climate change more to make up for other less climate-friendly actions they were undertaking.

The topics of “*social network initiation*” and “*awareness and fairness*” found at time 1 were not present at time 2 and time 3, and we consider that these topics could have been attached to events. In the case of “*awareness and fairness*,” we consider that the US campaign may have had an impact on the nature of this topic since the relevant topic words of this topic suggest users pointing out unconformity about individuals showing non-environmental behaviour (e.g., “*Like lecturing on climate change whilst having multiple homes, private jets, helicopters*—*private armed security you think rules do not apply to you*”). In addition, in times 2 and 3, when the pandemic was more spread across the globe, more restrictions were reducing the visibility of the non-environmental actions (e.g., travel on private planes). As for “*social network initiation*,” we consider that these were primarily calls for individuals to act on a particular event or to support a particular campaign or business; the lack of presence of this topic in time 2 and time 3 could also be due to the restrictions that governments enforced to achieve social distancing.

The topic “*awareness*,” which is present at all three time points, relates to the promotion of awareness around climate change. This topic seemed to change over time as well. At time 1 (3.7%), topic words included “award,” “elite,” “mention.” On further examination of tweets containing this topic, we found that the text of climate change mitigation was followed by the names of different international events; most of them related to climate change (e.g., “*… price in food and agriculture sciences …. and aims to improve crop production and hardiness helping to address challenges of global population growth and climate change…*,” “*I am sure many stars will use the Oscars to raise awareness of climate change*”). As for time 2 (2.3%) and time 3 (1.3%), the topic of “*awareness*” seemed to relate more to types of actions for climate change mitigation, topic encompassing topic words such as “packaging,” “bag,” “fair,” “env_conscious,” “raise_awareness,” “fundraising” (e.g., “*Young people all over the world are working hard to raise awareness of climate change …Climate change is real and we need to dramatically reduce greenhouse gas emissions*,” “*…environmentally conscious teen …who believes we can all continue to help the environment and remain sustainable even when we are stuck at home…*”). The change in this topic with the reduction in references to international events as a forum for raising awareness of climate change again may be attributed to the pandemic restrictions banning mass gatherings.

Our analysis also indicated a topic discussing what organisations do to combat climate change; we titled it “*Organisation’s Actions*.” At time 1 (1.8%), we found the discourse around this topic focussed on environmental actions taken, or to be taken in future by organisations, with relevant topic words: “reduce_passenger,” “green economy,” and “wildlife” (e.g., “*…Airlines may need to reduce passengers to take off due to climate change…”; “changing aircraft altitude could cut flights climate impact in half…*,” “*…how going digital and eradicating paper receipts sets companies on a sustainable path in the green economy…*”). For time 2 (0.9%) and time 3 (1.5%), other topic words emerged such as “bank,” “powerful,” “museum,” “amazon_pledge,” “cost_effective,” “airline,” and “hotel” (e.g., “*FYI systemic barriers to energy efficiency, low zero carbon world banks funding of high carbon polluting investments*”; “*Amazon pledges 2bn investment to fight climate change…*,” “*Alaska Airlines and Microsoft sign partnership to reduce carbon emissions with flights*”; “*Beer company… in Colombia introducing an Airbnb style platform, showcasing Colombian ecohotels that…sustainable tourism…*,” “*…redesign the museum to help bring about more equitable and sustainable futures in the climate change era, and then check out the design competition, Reimagining Museums for Climate Action…*”). The differences of the topic words between time points suggest that, whilst in time 1, people call for organisational actions, times 2 and 3 discussions were focussed on reporting organisational actions. This may reflect an increase in the importance placed upon corporate social responsibilities (Kolk and Van Tulder, 2010; Rosen-Zvi, 2011).

In addition, we found that the topic of “*collective actions*,” although present at all three time points, reduced its prevalence at time 2 (1.4%) and time 3 (1.4%) in comparison to its presence in time 1 (3%). One possible explanation for the low presence of this topic within the climate change mitigation discussion is the reduction of social behaviours due to the fear of the contagiousness of COVID-19. Moreover, as time passed, more restrictions were happening across the globe and, with it, the reductions in social and cooperative behaviour.

The topic of “*collective actions*” also appeared different in nature between times, with differences in relevant topic words evident. At the beginning of the year, at time 1, this topic included topic words such as “city,” “ready,” “milk,” “bee,” “collective,” “mobility,” and “network,” suggesting group behaviours (e.g., “*From banning pesticides to growing green roofs on bus stops, here are five ways communities are trying to save the bees….” It is a collective action to reduce the impact of climate change; we are making agriculture more efficient through AI, which is an important effort to make farming more sustainable,” “Sharing is caring. If we own less and use more things collectively …Sharing cars, exchanging clothes, lending, and borrowing tools*”). Relevant topic words within the same topic in time 2 were “threat,” “regulation,” “adapt,” “corona,” “cooperative,” and “electric_car,” suggesting a focus on individual behaviour that may benefit the community (e.g., “*…celebrates 26 years of unique hospitality…. the pioneer of eco-initiatives … lighting the path for a sustainable hospitality industry*,” “*Cooperatives for climate action…Heating, cooling, and planning for greener cities, sustainable transport…*,” “*build low-income housing for working families and or a nonprofit worker cooperative that gives back to the community through the arts sustainable farming…*”). For time 3, the most relevant terms of the topic “*collective actions*” were “chemical,” “bag,” “bond,” “solid,” “wealthy,” “attention,” “consume,” “tea,” “e-waste,” “evolution,” “socialism,” and “nitrogen,” suggesting cooperative behaviour to perform individually, e.g., (“*Cut the chemicals and learn how to create natural sustainable cleaning tools from things you will have in your cupboards*,” “*Sustainable behavior begins at home kick starts the International Ewaste Day caign[sic] by pledging to dispose off [sic] old electronics responsibly*…”).

We observed the emergence of new topics at time 2: “*food*,” “*nature conservation*,” and, at time 3, “*support farmers*,” and “*consequences of climate change solutions*.” For both times 2 and 3, we also observed the emergence of topics “*Eco-friendly products*,” “*sustainable production*,” “*innovative solutions*,” and “*third party actions*.” The topic “*support farmers*” at time 3 contained topic words related to the common agricultural policy (CAP), which suggest that the topic was based on opinions around the negotiations that happened in October at the European Council regarding the common agricultural policy reform package. This same event may have also influenced the discussion of other related topics such as “*eco-friendly products*,” “*sustainable production*,” and “*food*.”

The topic “*eco-friendly products*,” had similar topic words at both times 2 and 3, including “plastic,” “product,” “clothing,” “fashion,” “packaging,” and “sustainably”; and the topic “*sustainable production*” also presented similar topic words between times 2 and 3 such as “coffee,” “palm_oil,” “chocolate,” “manufacture,” “rainforest,” “dairy,” “bottle,” “beef,” “trading,” and “locally.” The topic modelling indicated these topics as different, and we interpreted them as such based on the conceptual differences between eco-friendly and sustainable products and the relevant topic words found for each topic. Notably, a product is eco-friendly when it has been designed to do the least possible damage to the environment (e.g., biodegradable plastic, a bamboo toothbrush), whereas sustainable products should recognise and minimise the social, ethical, and environmental impacts of the product; while sustainability may include eco-friendly activities, eco-friendly does not always translate to sustainable. It is unknown why these topics became more prevalent over time, but shopping and the nature of shopping may become more salient during the pandemic, given store closures, restrictions on purchasing, and the inability of many to visit shops due to shielding. The salience of purchasing behaviour may have been accompanied by an increased reflection on what kinds of products people were buying.

In addition, the topic of “*protests to government*” emerged in time 1 (1.5%) and time 2 (1.2%) only, with the promotion of active protesting to governments and organisations on climate change, for example, “dc_weekly” was a bigram found on tweets, encouraging people to go every week to protest about climate change at Washington DC. By time 3, this topic disappears; this, again, could be attributed to the restrictions on gatherings taking place in more countries or being out staged by other types of protests that happened at the time (e.g., protest anti-lockdown, or against face masks).

## Conclusion

According to the results of this study, public discourse around actions for climate change mitigation changed over time during the coronavirus pandemic on Twitter. We also observed an increase in the number of tweets that talk about actions to combat climate change at times 2 and 3 in comparison with time 1. With the use of Twitter data and machine learning techniques, we examined the public perceptions of actions for climate change mitigation during the coronavirus pandemic in 2020. Data indicate changes in perceptions over time, a pattern that appears to relate to restrictions on behaviour and social activity, and the increased salience of and potential reflection on certain behaviours. These changes support the idea that the pandemic impacted discourse around actions to tackle climate change. The development of sustainable communications and behavioural strategies could benefit from the findings of this paper in order to help support and promote those environmental behaviours that are likely to endure over time.

We observed changes in topics within tweets relating to climate change actions over time, which may reflect a pattern of change relating to restrictions on mobility and public gatherings due to COVID-19. We note a decrease in the prevalence of the “*awareness and fairness*” topic, possibly an incidental result of restrictions on mobility and people shielding to avoid COVID-19. In addition, the reduction of “*social network initiation*,” along with the change in nature of “*collective actions*” and “*awareness*” may be attributed to the same restrictions of mobility and gatherings. Over time, these appear to focus more on individual behaviour rather than group behaviour. These behaviours may change again once restrictions are lifted and individuals can travel, and meet socially, etc., without COVID-19 being a threat to their health. Changes here may, therefore, not persist long-term.

Changes in topics observed within tweets also indicated the possibility of increased salience of certain behaviour due to COVID-19. The increase of the prevalence of the “*environmental behaviour*” topic within tweets over time may be explained partially by an increased focus on certain environmental behaviour. It is possible that people had more time to reflect on their behaviour, particularly behaviour that given restrictions, which was also incidentally more environmentally friendly. On the other hand, this could be explained by people observing increases of non-environmental behaviour relating to measures to contain COVID-19 (e.g., face masks and single-use plastic) may also contribute to a drive to balance this by doing more for the environment (a compensatory effect). We may have observed a similar pattern in the increase in the prevalence of the topic of “*sustainable production*.” We suggest this may be the result of people’s reflections on their shopping habits. If changes in discourse we have observed are, indeed, due to increased salience of certain behaviours and increased reflection on environmental behaviour (cf. Rickard et al., 2014), then associated behavioural changes are likely to continue in the longer term. We note that information processing models [e.g., the Elaboration likelihood model (Petty and Cacioppo, 1986; Manca et al., 2020) and the Heuristic systematic model (Chen and Chaiken, 1999; Kim et al., 2015; Shi et al., 2020)] indicate that systematic consideration and elaboration of behaviour are likely to lead to more permanent behavioural changes. Manca et al. (2020) suggested that reflection on the consequences of unsustainable behaviour may be one main predictor of rational-intentional proenvironmental choice.

One of the most common themes observed around peoples’ perceptions of actions to mitigate climate change is “energy”; this did not change during the different time points, and it is consistent with previous findings in the literature (Kirilenko and Stepchenkova, 2014). This indicates that considering energy is the most discussed aspect of how to take action on climate change and may be the most salient to people.

Whilst coronavirus seemed to have an impact on the perceptions of actions for climate change mitigation, we observed that other unrelated events (e.g., general elections in the US, agricultural policies in the EU) also had an impact on the topics that emerged in the discussions of climate change mitigation. The topic of “*government actions*” was particularly frequently noted in the tweets around climate change mitigation for time 1; this is consistent with the literature that suggests climate change is a politicised issue (Weber, 2010; Clayton et al., 2015; Poortinga et al., 2019). However, these discussions seemed to be subdued at further time points, perhaps due to the presence of other issues, such as coronavirus; this seems to be consistent with findings in the literature, suggesting a “worry budget” and the coronavirus pandemic taking over political and administrative agendas (Bostrom et al., 2020; Helm, 2020).

The analysis performed here for the study of public perceptions of actions for climate change mitigation allowed us to evaluate the topics people talked about without predefined topics, contributing to the identification of topics that may not be considered in scientific reports of climate change mitigation (e.g., protests, nature conservation, collective action). It is important to note that whilst this research identified the topics people talked about when talking about climate change mitigation, we cannot define whether the discussions were positive or negative around a topic, for example, when we see terms like “carbon,” “emission,” “tax” on a topic, we can infer they talk about taxing carbon emissions, but we cannot define whether they are in favour or against of taxing carbon emissions. We also highlight that the classifications of action tweets for climate change mitigation were based on word coding from the researchers, and it is possible that words were identified, and, therefore, the related dataset could be slightly different if different researchers were to identify actions on climate change within tweets. Similarly, topics identified within topic analysis were interpreted and named by the researchers, and whilst we validated the topic interpretation with two different researchers, this is still open to subjective bias; notably, differences observed in themes over time are also dependent on the themes being identified as the same predominant types between time points, e.g., different types of tweets being identified as relating to “*collective action*” between time points may partly explain differences observed in this topic. We attempted to minimise subjective bias with second coding by additional researchers.

Whilst Twitter can be used to gather information about people’s perceptions of a topic, we acknowledge that the information found in the tweets does not necessarily define the views of the user. On Twitter, users self-present to a “networked audience,” an audience thought to consist of real and imaginary or potential viewers (Papacharissi, 2002). Moreover, it has been suggested that users write different tweets to different audiences; these tweets are typically influenced by the tweets, or retweets within the user’s feed (Marwick and Boyd, 2011). The implications of this for our data are that they may not represent people’s perceptions but may reflect what the user is exposed to on his or her news feed or virtual context and what he or she may perceive as “socially accepted” by other users.

Further research should focus on the evaluation of whether the topics we found in this study will still appear within public perceptions of climate change mitigation when the governments’ restrictions are lifted and COVID-19 becomes less of a threat to public health. We also consider it relevant to explore if the presence of one topic co-occurs with another topic, indicating whether specific discussions around actions for climate change mitigation prepare the ground for others; this could add to the study of behavioural spillover effects between environmental behaviours (Lanzini and Thøgersen, 2014; Thomas et al., 2016; Margetts and Kashima, 2017).

Overall, this paper highlights the importance of studying public perceptions of actions for climate change mitigation and how these are likely to change due to social context and events that may not be directly related to climate change, such as COVID-19. Previous literature suggests climate change and COVID-19 are similar global threats (Bostrom et al., 2020). This paper supports the idea that the coronavirus pandemic has impacted the nature of comments relating to actions for climate change mitigation on Twitter. We observed an increased number of tweets relating to actions to combat climate change over the course of the coronavirus pandemic as well as changes in the prevalence and types of topics emergent within tweets relating to action on climate change. Understanding perceptual and behavioural changes towards the environment due to COVID-19 and which of these are likely to persist in the long term could help in structuring environmental communications and behavioural interventions to support long-term sustainable behavioural change.

## Data Availability Statement

The raw data supporting the conclusions of this article will be made available by the authors, without undue reservation.

## Author Contributions

MGC, AS, and EF contributed to the conception, design of the study, and iterations of the manuscript revision. MGC wrote the first draft of the manuscript. VL and MGC contributed to the programming of the RNN and LDA models. MGC and AS performed the qualitative analysis. All authors contributed to the article and approved the submitted version.

### Conflict of Interest

The authors declare that the research was conducted in the absence of any commercial or financial relationships that could be construed as a potential conflict of interest.
