# Supplementary material for: Big Changes Start With Small Talk: Twitter and Climate Change in Times of Coronavirus Pandemic
Source: Front Psychol. 2021 Jun 15;12:661395. doi: 10.3389/fpsyg.2021.661395 (PMC8239357; doi:10.3389/fpsyg.2021.661395)
Supplement: Supplementary file 1 [file Data_Sheet_1.PDF]

Supplementary Material

1 Appendix. Supplementary tables and figures

1.1 Figures

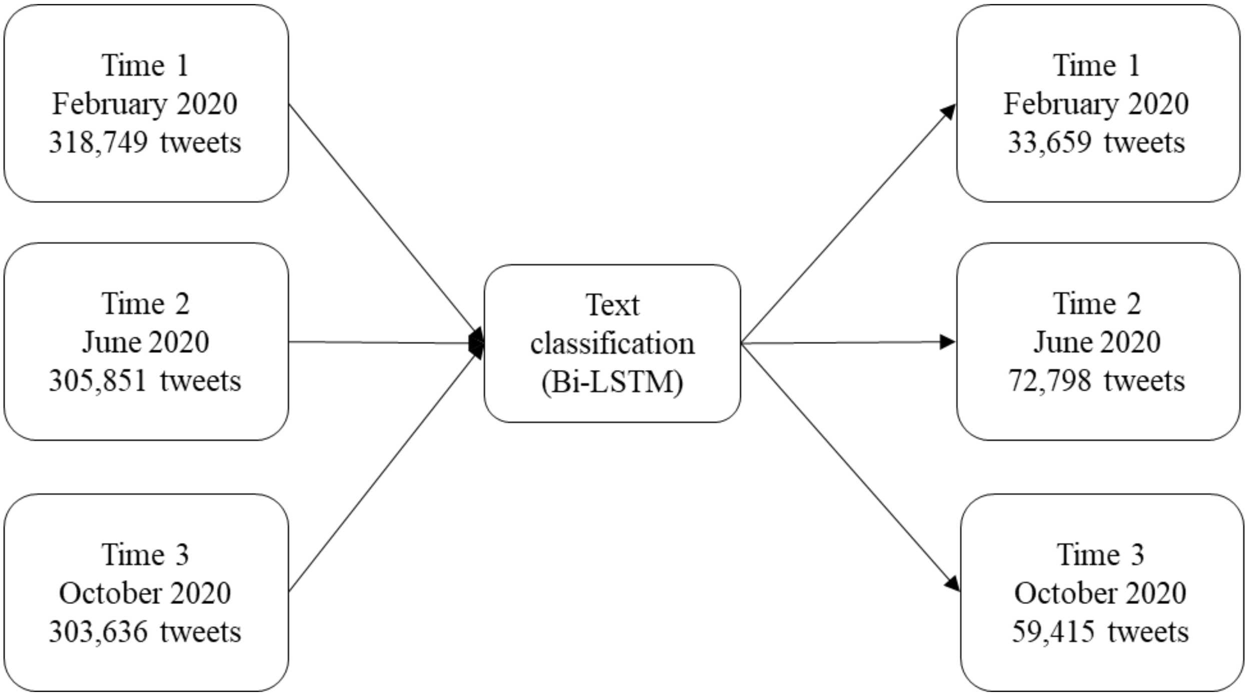

Figure 1. Total of streamed tweets and total of action tweets classified by the Bi-LSTM model for each timepoint

1.2 Tables

Table 1. Relevant topic words for each topic and their word counts

Time 1			Time 2			Time 3		
**Word**	**Topic**	**Word count**	**Word**	**Topic**	**Word count**	**Word**	**Topic**	**Word count**
carbon	1	3670	energy	1	5964	energy	1	6342
emission	1	2875	way	1	4027	food	1	5098
money	1	2119	people	1	3642	development	1	3619
fuel	1	1787	new	1	4793	future	1	3720
fund	1	1568	year	1	2362	way	1	3366
fossil	1	1387	emission	1	2453	new	1	3687
tax	1	1264	money	1	2176	solution	1	2907
people	1	2398	company	1	2985	people	1	3335

company	1	1171	time	1	2399	system	1	2688
address	1	1099	future	1	3943	industry	1	2644
energy	2	2265	development	2	4112	product	2	1998
people	2	2398	future	2	3943	material	2	940
food	2	1084	new	2	4793	packaging	2	704
help	2	1582	investment	2	2189	brand	2	760
renewable	2	832	economy	2	2186	plastic	2	1160
plan	2	913	energy	2	5964	fashion	2	665
new	2	1155	recovery	2	1614	cheap	2	428
car	2	925	job	2	2041	ecofriendly	2	505
clean	2	874	solution	2	2941	full	2	406
coal	2	910	goal	2	1871	design	2	707
award	3	58	product	3	2777	pay	3	330
liberal	3	79	plastic	3	1432	farmer	3	1297
trade	3	86	brand	3	1696	matter	3	180
mention	3	80	ecofriendly	3	886	true	3	204
direct	3	58	waste	3	1442	manager	3	183
third	3	43	help	3	2492	wood	3	279
essential	3	66	need	3	1186	nmore	3	120
div	3	62	material	3	1132	food	3	5098
pocket	3	74	fashion	3	1129	person	3	186
video	3	59	energy	3	5964	entrepreneur	3	193
city	4	417	food	4	4886	help	4	2160
ready	4	85	farmer	4	1289	store	4	306
milk	4	72	farming	4	973	ban	4	283
bee	4	81	agriculture	4	1154	dairy	4	1515
collective	4	54	system	4	2639	retailer	4	231
combat	4	940	farm	4	859	products_sold	4	206
customer	4	85	healthy	4	1626	gift	4	177
capital	4	60	meat	4	788	locally	4	2329
open	4	71	animal	4	731	beef	4	144
burn	4	71	production	4	1591	trading	4	154
wildlife	5	56	forest	5	660	plan	5	2530
decade	5	174	black	5	447	healthcare	5	276
skill	5	48	plant	5	1062	taxis	5	476
degree	5	50	electric	5	294	act	5	133
reduce_passenger	5	36	present	5	279	insurance	5	145
scientist	5	181	necessary	5	221	advantage	5	107
block	5	40	reliable	5	409	expertise	5	842
extraction	5	53	aware	5	200	build	5	387
takeoff_due	5	32	conservation	5	185	data	5	85
aircraft	5	38	specie	5	158	tax	5	726
town	6	59	packaging	6	354	profit	6	1428

run	6	56	bag	6	143	sign	6	1381
bullshit	6	39	fair	6	101	powered	6	1003
saving	6	55	range	6	95	theyll_plant	6	969
gold	6	34	person	6	93	woodendebitcard	6	961
hypocrisy	6	98	env_conscious	6	87	trees_plantchange	6	951
dc_weekly	6	20	aid	6	87	democracy	6	86
metal	6	33	consequences	6	83	taxpayer	6	98
show	6	85	collectively	6	81	reserve	6	75
radical	6	59	incentive	6	79	faster	6	83
tomorrow	7	69	massive	7	216	mining	7	94
platform	7	50	mining	7	239	interest	7	83
generate	7	31	milk	7	234	director	7	39
photo	7	26	scientist	7	194	foundation	7	34
actor	7	25	circular	7	144	make	7	32
show	7	85	designer	7	201	electric_car	7	27
solid	7	26	core	7	135	australian	7	22
burning	7	28	situation	7	116	vaccine	7	19
sweater	7	31	caign	7	122	film	7	18
positively	7	15	metal	7	111	renewable	7	15
twitter	8	48	economically	8	38	method	8	278
crazy	8	44	phase	8	37	forever	8	157
hot	8	44	cycling	8	37	cost_effective	8	198
decent	8	25	summit	8	33	poor	8	257
push	8	57	working	8	31	fabric	8	136
find	8	17	vote	8	29	rapid	8	84
bicycle	8	25	textile	8	29	metal	8	96
httpstco	8	17	auspol	8	16	lack	8	85
technew	8	15	timber	8	11	innovator	8	73
helicopter	8	45	action_climate	8	10	chemical	9	232
hydrogen	9	78	threat	9	178	bag	9	225
climateactnow_auspol	9	50	regulation	9	179	bond	9	176
globalwarming	9	24	boost	9	169	solid	9	116
utility	9	63	category	9	86	wealthy	9	122
visit	9	25	joint	9	97	attention	9	110
guitar	9	28	code	9	120	ewaste	9	111
court	9	34	adapt	9	105	machine	9	67
persuade	9	13	hospital	9	106	socialism	9	66
art	9	26	shoe	9	108	consume	9	41
mansion	9	82	cooperative	9	61	coffee	10	197
			importance	10	254	income	10	663
			coffee	10	227	cover	10	155

palm_oil	10	108	minimum	10	140
recycled	10	166	interested	10	309
push	10	93	realistic_source	10	76
final	10	64	raise_awareness	10	62
death	10	80	code	10	75
chion	10	82	rent	10	60
rent	10	89	trash	10	43
trip	10	81	destruction	11	93
bank	11	270	engine	11	102
powerful	11	114	inclusion	11	74
comprehensive	11	108	island	11	58
lab	11	92	integration	11	61
figure	11	83	diesel	11	67
easily	11	86	disease	11	71
fine	11	122	trial	11	64
win	11	130	plate	11	57
rapidly	11	82	planting	11	81
criterion	11	70			
aim	12	164			
park	12	134			
lesson	12	120			
leather	12	97			
room	12	89			
lol	12	48			
reasonable	12	56			
grateful	12	59			
agile	12	39			
toilet	12	38			

2 Appendix: Source codes in Python for Bi-LSTM and topic modelling

2.1 Bi-LSTM in Python

[]:

```
import pandas as pd
import numpy as np

from__future______import print_function, division
from builtins import range

   import osimport sys
 import numpy as
 npimport pandas
 as pd
 import matplotlib.pyplot as plt

 from keras.preprocessing.text import Tokenizer #tokenization: turn a sentence into a list
 of strings.

 from keras.preprocessing.sequence import pad_sequences

 from keras.layers import Dense, Input, GlobalMaxPooling1Dfrom
 keras.layers import LSTM
 from keras.layers import Convolution1D,
 GlobalMaxPooling1D,GlobalAveragePooling1D,GlobalMaxPool1D
 from keras.layers import Conv1D, MaxPooling1D, Embeddingfrom
 keras.models import Model
 from sklearn.metrics import roc_auc_score
 from keras.layers.wrappers import TimeDistributed, Bidirectionalfrom
 keras.layers.normalization import BatchNormalization
 from keras import backend as K
 from keras.layers import Convolution1D, GlobalMaxPooling1D,.
  ↪GlobalAveragePooling1D,GlobalMaxPool1D

  from keras.layers import GlobalMaxPooling1D, Conv1D, MaxPooling1D, Flatten, Bidirectional,
                             SpatialDropout1D, concatenate,Concatenate
```

[ ]:

```
#   configuration

MAX_SEQUENCE_LENGTH = 300 # you can set this by loading al the data and
choosing the max sequence or lokking at a histogram of each comment length

MAX_VOCAB_SIZE = 20000 # A NATIVE ENGLISH SPEAKER USES ONLY AROUND 20000
WORDS AND IT'S REASONABLE TIME/SIZE TO ANALYIS

EMBEDDING_DIM = 300 # THE SIZE OF EACH WORD VECTOR- PRETRAINED VECTORS
ONLY COME.

 IN SPECIFIC SIZES
```

[ ]:

```
#LOAD INTO PRE-TRAINED VECTORS

print('Loading word vectors...') word2vec
= {}
with open(os.path.join(r'C:\\glove.6B.300d.txt'),encoding="utf8").
  ↪as f:

     for line in f:
          values = line.split()
          word = values[0]
          vec = np.asarray(values[1:], dtype = 'float32')
          word2vec[word] = vec
print('Found %s word vectors' % len(word2vec))
```

[ ]:

```
train = pd.read_csv(r'C:\\labelled tweets_for RNN.csv')

sentences = train['x'].fillna('DUMMY_VALUE').values
```

[ ]:

```
#sentences

possible_labels = ['Label']
targets = train[possible_labels].values
# convert the sentences (strings) into integers
tokenizer=Tokenizer(num_words=MAX_VOCAB_SIZE
) tokenizer.fit_on_texts(sentences)

sequences=tokenizer.texts_to_sequences(sentences)
```

[ ]:

```
#get word - index mapping

word2idx = tokenizer.word_index # word_index connects the word and the index from
embedding

print('Found %s unique tokens' % len(word2idx))
```

[ ]:

```
data = pad_sequences(sequences, maxlen = MAX_SEQUENCE_LENGTH) # padding to
make sure that the documents have the same size
```

[ ]:

```
print("Shape of data tensor:", data.shape) # (num of sequences, sequence length )
```

[ ]:

```
# prepare embedding matrix

print("Filling pre-trained embeddings...") num_words =
min(MAX_VOCAB_SIZE, len(word2idx) + 1)
num_words
```

[ ]:

```
embedding_matrix = np.zeros((num_words,
EMBEDDING_DIM))for word, i in word2idx.items():
    if i < MAX_VOCAB_SIZE:
        embedding_vector = word2vec.get(word)if
        embedding_vector is not None:
          # words not found in embedding index will be all zeros.

          embedding_matrix[i] = embedding_vector
```

[ ]:

```
embedding_layer = Embedding(
  num_words,
  EMBEDDING_DIM,
  weights=[embedding_matrix],
  input_length=MAX_SEQUENCE_LENGTH,
  trainable=False)
```

[ ]:

```
from sklearn.preprocessing import LabelEncoder
encoder = LabelEncoder()

targ = train["Label"]

targ_encoded = encoder.fit_transform(targ)
train["targ"] = targ_encoded

possible_labels = ["targ"]

targets = train[possible_labels].values

from sklearn.model_selection import train_test_split

train.describe()
```

[ ]

```
print('Building model...')

# train a 1D convnet with global maxpooling
input_ =
Input(shape=(MAX_SEQUENCE_LENGTH,))x
= embedding_layer(input_)

x = Bidirectional(LSTM(15, return_sequences = True))(x)x =
GlobalMaxPool1D()(x)
output = Dense(len(possible_labels), activation = 'sigmoid')(x) # the last activation has
to be sigmoid because it's classification
model = Model(input_, output)
model.compile(
    loss='binary_crossentropy',
    optimizer='rmsprop',
    metrics=['accuracy'])
print('Training model...')
r = model.fit(
    X_train,
    y_train,
    batch_size=BATCH_SIZE,
```

[ ]:

```
output = model.predict(X_test)
np.mean((output >= 0.5) == y_test)
```

[ ]:

```
# DEPLOY

df = pd.read_csv(r'C:\\ tweet_forRNN.csv') df.head()
```

[ ]:

```
sentences1 = df['x'].fillna('DUMMY_VALUE').values
sequences1 = tokenizer.texts_to_sequences(sentences1)
```

[ ]:

```
print("max sequence length:", max(len(s) for s in sequences1))
print("min sequence length:", min(len(s) for s in sequences1)) s1 =
sorted(len(s1) for s1 in sentences1)

print("median sequence length:", s1[len(s1) // 2])
```

[ ]:

```
data1 = pad_sequences(sequences1, maxlen = MAX_SEQUENCE_LENGTH)
```

[ ]:

```
print('Shape of data tensor:', data1.shape)
```

[ ]:

```
p = model.predict(data1)
df['prediction'] = p

s = df.head(1000)
```

2.2 Topic modelling with genism in Python

[ ]:

```
from os import path
import matplotlib.pyplot as plt
import seaborn as sns
import numpy as
npimport pandas
as pd
import matplotlib.pyplot as plt
%matplotlib inline
import pickle
```

[ ]:

```
#Gensim

import gensim
import gensim.corpora as corpora
from gensim.utils import simple_preprocessfrom
gensim.models import CoherenceModel
from sklearn.feature_extraction.text import TfidfTransformer
import re
import numpy as
npimport pandas
as pd
from pprint import pprint
#spacy is used for the lemmatization

import spacy
```

[ ]:

```
#import data

df=pd.read_csv(r"C:\\Users\\actiontweets_for Topicmodelling.csv") df.head()

datatmp = df.values.tolist()
data= datatmp
```

[ ]:

```
#Preparing stopwords


#NLTK stopwords

from nltk.corpus import stopwords
stop_words= stopwords.words("english")
stop_words.extend(["climate","action","s
ure","support","fight","https","commit","
","change","smart", "move","save",
"planet","global",
"warming","be","httpstcox", "httpstcoy",
"green",.
 ↪"fuck","httpstcoz", ".","  _ ", "good", "condom", "something", "someone",.
 ↪"anything", "lot", "bit", "anyone",
 "really" range(0,1000),"realdonaldtrump","amp","nee","action","impact","httpst
 cod","bitch","sotu" ,climatechange","news","bro","tout","httpscoi","email","ht
 tpstcoz","changea"])
```

[ ]:

```
#Tokenization of words and clean-up text

def
    sent_to_words(sentence
    s): for sentence in
    sentences:
        yield(gensim.utils.simple_preprocess(str(sentence), deacc=True))
        #deacc=True removes punctuations

data_words=list (sent_to_words(data))
```

[ ]:

```
#Creating bigram and trigram model


#Build the bigram and trigram model

bigram_mod=
gensim.models.phrases.Phraser(bigram)
trigram_mod=
gensim.models.phrases.Phraser(trigram)
```

[ ]:

```
#Define functionsfor stopwords

def remove_stopwords(texts):
    return [[word for word in simple_preprocess(str(doc)) if word not in.
 ↪stop_words] for doc in texts]
#for bigrams

def make_bigrams(texts):
    return[bigram_mod[doc]for doc in texts]

#for lemmatization

def lemmatization(texts, allowed_postags=["NOUN","ADJ","ADV"]):
    """https://spacy.io/api/annotation"""

    texts_out= []
    for sent in texts:
        doc=nlp("
        ".join(sent))
        texts_out.append([token.lemma_ for token in doc if token.pos_
        in.allowed_postags])
        return
     texts_out
```

[ ]:

```
#remove stopwords

data_words_nostops=remove_stopwords(data_words)

#form bigrams

data_words_bigrams=make_bigrams(data_words_nostops)

#form trigrams

data_words_trigrams=make_trigrams(data_words_nostops)

#Initialize spacy "en" model, keeping only tagger component for
efficiency #python3 -m spacy download en

nlp=spacy.load("en_core_web_sm", disable=["parser", "ner"])

#lemmatization keeping only noun, adj, vb, adv bigrams

data_lemmatized= lemmatization(data_words_bigrams,.
 ↪allowed_postags=["NOUN","ADJ","ADJ"])

print(data_lemmatized[:18])
```

[ ]:

```
#Creating dictionary and corpus neded for the topic modeling the two main
inputs.
 for LDA

id2word=corpora.Dictionary(data_lemmatized)

#create the corpus. The produced corpus is a mapping of [word_id,.
 ↪word_frequency] This is the imput by the LDA model
```

[ ]:

```
texts=data_lemmatized
#Term Document Frequency

corpus=[id2word.doc2bow(text)for text in texts]
#view

print(corpus[:5])#gensim creates a unique id for each word in the document.
```

[ ]:

```
##Build the model

#time1

lda_model = gensim.models.ldamodel.LdaModel(corpus=corpus, id2word=id2word,.
  ↪num_topics=9, alpha="asymmetric", eta=0.91,per_word_topics=True)
#time 2

lda_model = gensim.models.ldamodel.LdaModel(corpus=corpus, id2word=id2word,.
  ↪num_topics=12, alpha="asymmetric", eta=0.91,per_word_topics=True)
#time 3

lda_model = gensim.models.ldamodel.LdaModel(corpus=corpus, id2word=id2word,.
  ↪num_topics=11, alpha="asymmetric", eta=0.91,per_word_topics=True)
```

[]:

```
# Computing perplexity and coherence
score

#Compute perplexity

print("\nPerplexity:", lda_model.log_perplexity(corpus))

#Compute coherence score

coherence_model_lda = CoherenceModel(model=lda_model, texts=data_lemmatized,
dictionary=id2word, coherence="c_v") coherence_lda =
coherence_model_lda.get_coherence()print("\nCoherence Score: ",
coherence_lda)
```
